# Case Report: Off-label treatment of idiopathic hypereosinophilic syndrome with Omalizumab

**DOI:** 10.3389/fphar.2023.1095737

**Published:** 2023-06-08

**Authors:** Zhiyu Zhang, Yingxin Sun, Su-Ning Chen

**Affiliations:** ^1^ Department of Hematology, First Affiliated Hospital of Soochow University, Suzhou, China; ^2^ Jiangsu Institute of Hematology, Suzhou, China; ^3^ National Clinical Research Center for Hematologic Diseases, Suzhou, China

**Keywords:** omalizumab, idiopathic hypereosinophilic syndrome, eosinophilia, steroid resistance, case report

## Abstract

Idiopathic hypereosinophilic syndrome (IHES) is a rare disease characterized by causeless persistent hypereosinophilia and eosinophilia-associated end-organ damage. Current treatment modalities don’t meet the needs due to adverse events of steroids as first-line therapy and the limited efficacy of second-line treatments, underscoring the need for new therapeutic strategies. Here we presented two cases of IHES with different clinical manifestations that were both refractory to corticosteroids. Patient #1 experienced rashes, cough, pneumonia, and steroid-induced side effects. Patient #2 had severe gastrointestinal symptoms attributed to hypereosinophilia. They both had high levels of serum IgE, didn’t respond well to second-line treatments of interferon-α (IFN-α) and imatinib, and Mepolizumab was not accessible. We then innovatively switched to Omalizumab, an anti-IgE monoclonal antibody approved for allergic asthma and chronic idiopathic urticaria. Patient #1 was treated with Omalizumab 600 mg per month for 20 months; his absolute eosinophil count (AEC) decreased significantly and has stabilized at around 1.0×10^9^/L for 17 months, with complete relief from erythra and cough. Patient #2 recovered promptly from severe diarrhea with a sharp drop in AEC after 3 months of treatment with omalizumab at 600 mg per month. Therefore, we concluded that Omalizumab may be a seminal therapeutic strategy for IHES patients who are refractory to corticosteroids, whether as long-term management of AEC or as an urgent intervention to address severe symptoms caused by eosinophilia.

## Introduction

Eosinophilia and related syndromes are a group of heterogeneous disorders characterized by persistent eosinophilia in the peripheral blood (PB) or tissue, with or without eosinophil-mediated end-organ damage. Hypereosinophilia (HE) is defined as an AEC above 1.5×10^9^/L or tissue eosinophilia, including hereditary HE, primary HE, reactive HE, and HE of undetermined significance, also known as idiopathic HE (Ali et al, 2021). Hypereosinophilic syndrome (HES) refers to HE variants with end-organ damage caused by eosinophilia. The term idiopathic hypereosinophilic syndrome (IHES) is used when associated organ damage has no identifiable etiologies, with AEC >1.5×10^9^/L for at least 6 months (Shomali and Gotlib, 2022).

Corticosteroids are the first-line therapy for HES, and high-dose corticosteroid therapy (1 mg/kg prednisone to 1 g prednisolone) is recommended as an urgent intervention when symptoms of end-organ damage are present or suspected. Second-line treatments include hydroxyurea, IFN-α, imatinib, and Mepolizumab (Klion, 2015). A retrospective study assessed and compared the effectiveness of current HES treatments (Ogbogu et al, 2009). In this study, 85% of patients initially treated with corticosteroids achieved complete or partial remission. The complete/partial response rates of hydroxyurea and IFN-α were 72% and 50% respectively. However, these treatments may lose effectiveness and non-negligible side effects may occur over time. Therefore, steroids were discontinued in 42% of patients and the discontinuation of hydroxyurea and IFN-α was more frequent (77% and 87%). The response rate of imatinib was only 23% in *PDGFRα*-negative HES patients. Mepolizumab, a monoclonal antibody targeting interkulin5 (*IL-5*), could control AEC, relieve disease symptoms, and reduce the prednisone dosage in HES patients (Roufosse et al, 2020; Alves Júnior et al, 2021; Pane et al, 2022). Nonetheless, its high cost limits its application. There are no recommendations for alternative treatment modalities for HES when steroid therapy has failed. As a result, treatment needs are largely unmet. Omalizumab is an anti-IgE monoclonal which the Food and Drug Administration has approved for allergic asthma, chronic idiopathic urticaria, and nasal polyps. In addition to the above three indications, the European Medicines Agency also approved it for chronic rhinosinusitis with nasal polyps. In China, indications approved by the National Medical Products Administration (NMPA) only include allergic asthma and chronic idiopathic urticaria. It has also been used successfully as an off-label treatment for eosinophilic pneumonia and eosinophilic otitis media (Okude et al, 2012; Laviña-Soriano et al, 2017). Here, we reported two patients with IHES who were treated effectively with Omalizumab.

## Case description

### Case 1

**FIGURE 1:**
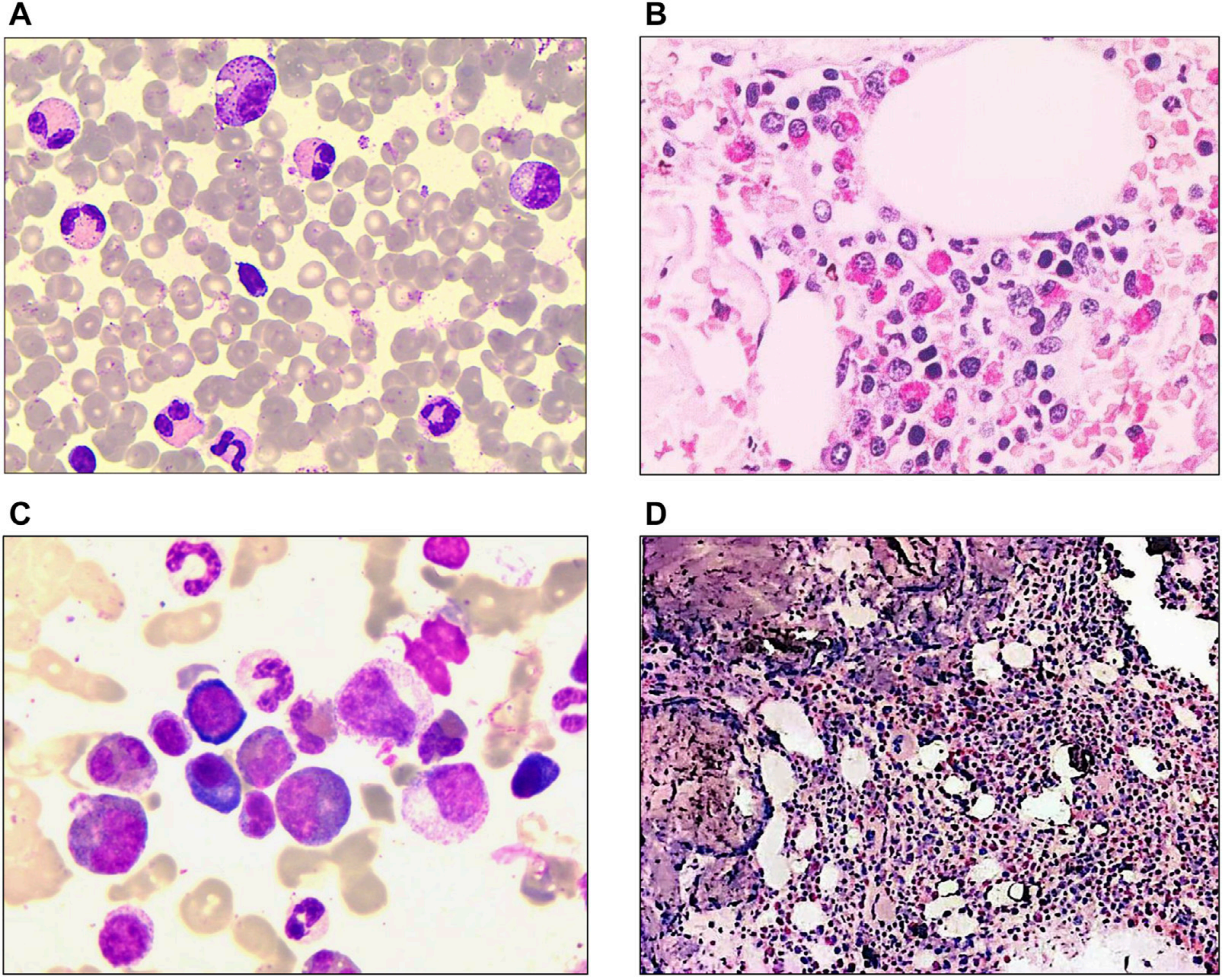
Laboratory characteristics of the patients at diagnosis. **(A)** Morphological analysis of patient #1 revealed bone marrow with 23.5% eosinophils and granulocytic proliferation (Wright-Giemsa staining ×1000). **(B)** Bone marrow biopsy of patient #1 showed hematopoiesis with marked eosinophil infiltration (Hematoxylin-eosin staining ×400). **(C)** Bone marrow morphology of patient #2 showed hypercellular marrow with marked eosinophilia (41% eosinophils) (Wright-Giemsa staining ×1000). **(D)** Bone marrow biopsy of patient #2 revealed granulocytic proliferation and significantly increased eosinophils. (Hematoxylin-eosin staining ×100).

In February 2019, a 48-year-old man visited our department, complaining of multiple pruritic rashes on limbs with cough and expectoration for 1 week. Both lower limbs displayed continuous brown lesions, with a scattered distribution on the upper limbs. A chest computed tomography (CT) scan showed inflammation and bulla on both lungs. Bloodwork revealed PB eosinophilia (AEC, 6.26 × 10^9^/L; 42.1% eosinophils), with normal hemoglobin and platelet counts. Lactic dehydrogenase (LDH) was 277 U/L and serum IgE was 579.8IU/mL (Supplementary Table S1). A morphologic review of bone marrow aspirate showed granulocytic proliferation, with 23.5% eosinophils (Figure 1A). Flow cytometric immunophenotyping and bone marrow biopsy confirmed eosinophilia (Figure 1B). *PDGFRA, PDGFRB, JAK2, FGFR1*, and *BCR::ABL1* rearrangement were not detected. We also conducted tests for C reactive protein (CRP), procalcitonin (PCT), parasite infection, fungal infection, allergen, autoantibodies, tumor markers, and thyroid function, the outcomes of which were all normal. We examined the specific IgG and IgE antibody to Aspergillus fumigatus in serum and it was negative. Considering the negative result, no symptoms of asthma, and nonspecific imaging features, we also ruled out the diagnosis of allergic bronchopulmonary aspergillosis (ABPA). Moreover, the patient did not take medications that might lead to eosinophilia. Integrating clinical characteristics, laboratory parameters, and unexplained eosinophilia, we attributed his rashes and cough to eosinophilia and made the diagnosis of HES.

**FIGURE 2:**
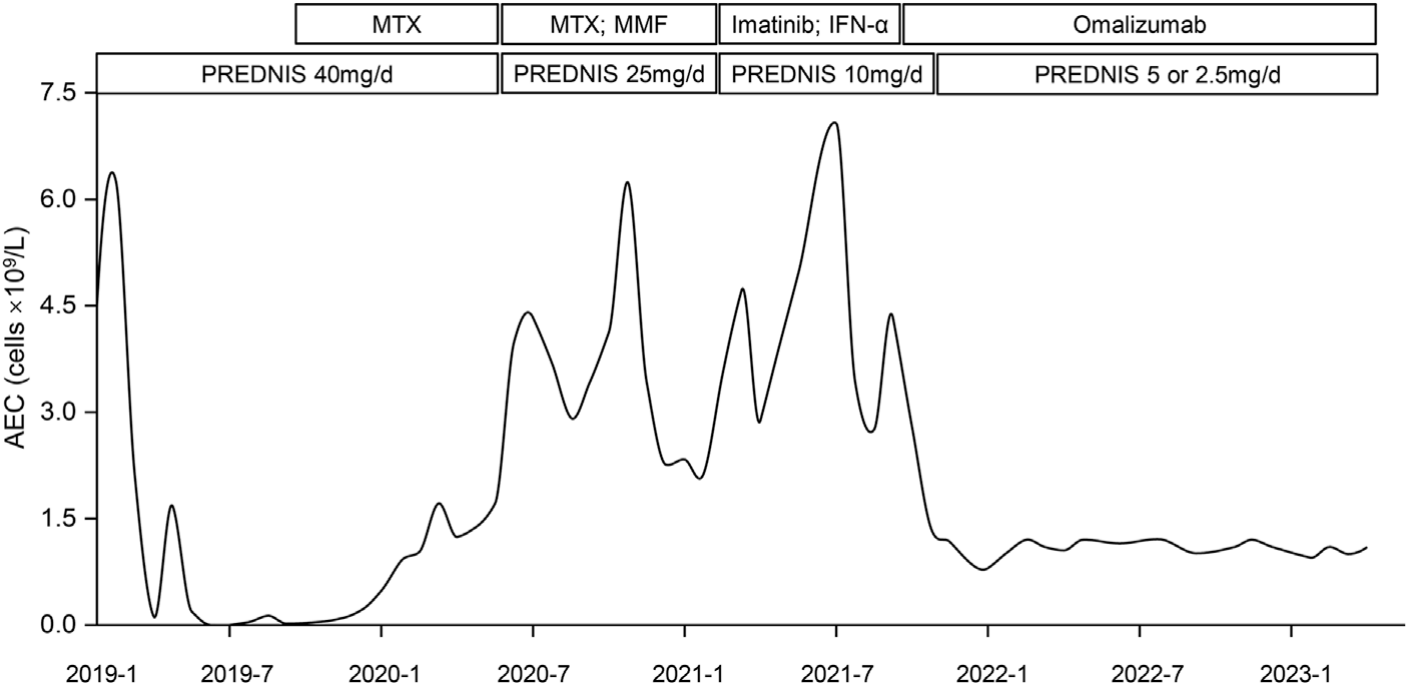
Variation curve of patient #1’s absolute eosinophil count (AEC) over time and different treatments. The curve demonstrated the change of the patient’s PB eosinophil count over time and adjustments of therapeutic regimen. It declined rapidly after first initiation of prednisolone (PREDNIS) and rose gradually since January 2020. Application of methotrexate (MTX), mofetil tablets (MMF), Imatinib, and Interferon-α (IFN-α) did not bring down the AEC. Omalizumab was used in September 2021, after which his PB eosinophil count decreased rapidly and reached 0.8 × 10^9^/L in January 2022. It has stayed around 1.0 ×10^9^/L for 17 months. Prednisolone was tapered to 2.5 mg/d.

He was treated with high-dose corticosteroids (prednisolone 40 mg/d) since February 2019. The rashes faded away and AEC returned to normal in 1 month (Figure 2). However, tapering of the prednisolone dose resulted in a relapse of rashes; the dose was thus kept at 40 mg/d, with the addition of methotrexate (MTX) tablets. Despite his AEC dropping to normal, his LDH kept rising and reached 752.7 U/L in July 2019 (Supplementary Table S1). In August 2019, the patient was retreated for pneumonia, accompanied by high fever, pruritus rashes, and cough. A chest CT scan revealed both lungs with inflammation and consolidation. The CRP and PCT were normal, and all etiological examinations showed negative results. He was treated with linezolid and biapenem for 1 week with little success until the addition of higher dose glucocorticoids (prednisolone 60 mg/d) resulted in a normal temperature and resolution of lung inflammation. Unfortunately, he did not undergo a bronchoalveolar lavage fluid test or transbronchial lung biopsy to confirm eosinophil infiltration in his lungs. However, considering that glucocorticoids improved pulmonary symptoms, we presumed the symptoms were caused by HE. After recovery from pneumonia, he continued with prednisolone of 25 mg/d with MTX and mycophenolate mofetil tablets (MMF) since August 2020. Frustratingly, his AEC began to rise again and exceeded the normal range in January 2020 (Figure 2). In October 2020, it rose to 6.24× 10^9^/L, with skin rashes and persistent cough, followed by bilateral osteonecrosis of femoral heads. Based on his unexplained eosinophilia for more than 6 months and symptoms attributed to it, we modified the diagnosis to IHES. Due to steroid-induced adverse events, we cut down the prednisolone to 10 mg/d and successively administered imatinib and IFN-α, but all yielded unsatisfactory results. His AEC was as high as 7.07×10^9^/L in July 2021 (Supplementary Table S1), and his rashes also worsened.

Given the patient’s financial burden, Mepolizumab was removed from candidacy, and Omalizumab was considered after reexamination of serum total IgE (500IU/mL). With informed consent, the patient received a monthly injection of Omalizumab 600 mg with prednisolone 10 mg/d since September 2021, and he has been treated 20 times with Omalizumab to date. His rashes and cough recovered with no visible adverse reactions. His AEC was examined monthly since the first injection. As is shown in Figure 2, it decreased from 4.31 × 10^9^/L to 2.78 × 10^9^/L after the first month. It dropped to 0.8 × 10^9^/L in January 2022 and has been maintained around 1.0×10^9^/L for 17 months. His LDH also reduced to normal (193U/L in March 2022). During the administration of Omalizumab, the dose of prednisolone was gradually reduced to 2.5 mg/d and no symptomatic relapses were noted.

### Case 2

A 29-year-old male patient was hospitalized in March 2017 with a fever up to 40°C and prolonged PB eosinophilia for 5 years. Bloodwork showed an AEC of 7× 10^9^/L and, as is shown in Figure 1C, bone marrow smears revealed hypercellular morphology with marked eosinophilia (41% eosinophils). Flow cytometric immunophenotyping and bone marrow biopsy confirmed eosinophilia (Figure 1D). While ruling out potential secondary causes for eosinophilia, we detected no gene rearrangements, karyotype abnormalities, or mutations associated with myeloid malignancies. Hence, we made a diagnosis of idiopathic HE.

He was initially treated with prednisolone (40 mg/d and gradually tapered off) for 9 months until January 2018; his AEC decreased and was maintained around 1.5× 10^9^/L. After the withdrawal of steroids, AEC was not monitored until September 2020, when he was hospitalized again with fever, lymphadenectasis, abdominal pain, continuous diarrhea, and AEC of 25.83× 10^9^/L. The patient refused endoscopic biopsy and we found no others causes for gastrointestinal symptoms, which may be related to eosinophilia. Prednisolone up to 40 mg/d could not control his AEC and symptoms. Then we adjusted to imatinib and IFN-α since November 2021; his AEC reduced to 7.01× 10^9^/L but severe gastrointestinal symptoms did not resolve. With informed consent, he received Omalizumab injection three times (600 mg at a time) in January, February, and March 2022. His serum IgE was 1990 IU/mL before the first injection. His AEC dropped to 2.23× 10^9^/L after 3 injections, and diarrhea was also relieved. Therefore, we considered the diarrhea to be caused by HE and modified the diagnosis to IHES.

## Discussion

IHES is a rare non-clonal hypereosinophilic syndrome with unknown etiology. Although glucocorticoids are an accepted first-line treatment strategy, there is insufficient evidence on the optimal therapeutic strategies for patients with glucocorticoid resistance. Here, we reported two IHES cases.

Unexplained AEC in patient #1 remained at more than 1.5 × 10^9^/L for 6 months with pneumonia. Patient #2 presented with causeless HE for more than 6 months with gastrointestinal symptoms. They both achieved temporary remission after corticosteroid therapy, but the diseases recurred under sustained corticosteroid therapy, indicating steroid resistance. We tried multiple second-line therapies for them, but the results were disappointing. Mepolizumab has been proven to be efficient in IHES in several studies, and has been approved by the FDA and EMA for hypereosinophilic syndrome (Shomali and Gotlib, 2022). However, as a novel agent, it’s highly expensive and unaffordable for our patients, and we needed to explore alternatives.

Omalizumab is a humanized anti-IgE monoclonal antibody, and it is 10 times cheaper than Mepolizumab. Chinese NMPA had approved it for treatment of allergic asthma and chronic idiopathic urticaria. Previous studies showed that Omalizumab could decrease AEC in allergic asthma patients (Massanari et al, 2010) and relieve the symptoms of hypereosinophilic asthma, especially for severe cases that cannot be controlled by traditional treatments (Koski and Grzegorczyk, 2020). For off-label uses, it was effective in eosinophilic pneumonia with poor response to steroids (Domingo and Pomares, 2013; Laviña-Soriano et al, 2017). Moreover, a study by Grieco et al reported that Omalizumab successfully treated a chronic spontaneous urticaria patient with the comorbidities of ulcerative colitis and IHES who was unresponsive to anti-histamines and steroids (Grieco et al, 2018). In addition, studies found PB eosinophil and serum IgE levels were predictors of Omalizumab efficacy in asthma (Sheehan et al, 2020). Although higher IgE (more than 350IU/mL) and AEC (more than 3×10^9^/L) were associated with a greater risk of disease progression, the risk was reduced by 59% after Omalizumab treatment, while patients with lower AEC were almost unresponsive to Omalizumab (Busse et al, 2013). Recently, a retrospective study reported six IHES patients receiving Omalizumab with a low hematologic remission rate (Chen et al, 2022). However, the patients in their study were not assessed for serum IgE levels before Omalizumab treatment, and Omalizumab was approved for allergic asthmatics with an elevated serum IgE level (>30 IU/mL). We conjectured based on historical data that higher IgE levels might be associated with better efficacy of Omalizumab in IHES and so included Omalizumab as an option for our patients (Busse et al, 2013; Sheehan et al, 2020; Chen et al, 2022).

The mechanism of Omalizumab on eosinophils could be both direct and indirect. It could directly bind to the FcɛRI receptors on eosinophils to induce apoptosis (Metz et al, 2017). It could also bind to free IgE to prevent its link to IgE receptors on eosinophils, reducing eosinophil activation and downregulating the IgE receptors (Kaplan et al, 2017). Meanwhile, it could bind to receptors on Th2 cells to inhibit the release of interleukin 4, 5, and 13, all of which were responsible for eosinophil recruitment and activation (Coyle et al, 1996).

Therefore, we finally switched to Omalizumab. A total of 600 mg Omalizumab was injected subcutaneously in two injections once time per month, which is convenient for patients. And no side effect was noticed in both patients, indicating the safety of using Omalizumab at this dose and frequency. Patient #1’s AEC decreased rapidly since the first injection, reached a bottom of 0.8×10^9^/L, and then stabilized at around 1.0×10^9^/L (Figure 2), which is lower than the World Health Organization’s suggested threshold for starting treatment (Shomali and Gotlib, 2022). Even though the dose of prednisolone was tapered during Omalizumab treatment, his rashes and cough continued to improve and were cured. Patient #2 only received Omalizumab injection for 3 months,; his severe diarrhea recovered soon after and his AEC also decreased significantly. Thus, we suggested Omalizumab as a second-line treatment option for IHES, especially in patients with high serum IgE levels who were refractory to glucocorticoids and require management of AEC or emergency intervention for severe symptoms. It should be noted that, although we used Omalizumab 600 mg monthly, the appropriate dose of it in IHES needs to be confirmed in prospective clinical trials.

Although we successfully treated two IHES patients with Omalizumab, this work also has some limitations. First, as a case report, it is not enough to accurately assess the general response of IHES to Omalizumab. Second, the relatively short follow-up time limits the ability to evaluate the duration of Omalizumab efficacy. Third, our result is inconsistent with some of the former studies, and the efficacy of Omalizumab on IHES needs further validation. Nevertheless, our work adequately demonstrated the potential of Omalizumab in controlling eosinophil levels and relieving IHES symptoms.

## Conclusion

We reported two cases of steroid-refractory IHES treated with Omalizumab; in both cases Omalizumab effectively reduced the AEC and alleviated the end-organ damage caused by eosinophilia. Consequently, we believe that Omalizumab may be a safe and promising option as a second-line therapy in IHES, both in the management of AEC and in the emergency intervention of hypereosinophilia-related symptoms, especially in patients refractory to glucocorticoids (El-Qutob, 2016).

## Data Availability

The original contributions presented in the study are included in the article/Supplementary Material, further inquiries can be directed to the corresponding author.

## Supplementary material

SUPPLEMENTARY TABLE S1 Detailed information of patient #1’s blood eosinophil count and Lactic dehydrogenase level upon every test. The first three columns of the table demonstrate the accurate values of the patient’s blood eosinophil count and percentage of eosinophils every time he had a blood routine examination (a total of 75 times) since his first visit. The next two columns show the exact values of his Lactic dehydrogenase level every time he tested it (a total of 15 times) throughout the whole course of the disease.
